# The Case for Octopus Consciousness: Valence

**DOI:** 10.3390/neurosci3040047

**Published:** 2022-11-17

**Authors:** Jennifer Mather

**Affiliations:** Department of Psychology, University of Lethbridge, Lethbridge, AB T1K 3M4, Canada; mather@uleth.ca

**Keywords:** octopus, consciousness, valence, cephalopod

## Abstract

Octopuses may demonstrate perceptual richness, neural unity, temporality, and finally, valence or affective evaluation, as the neural basis for consciousness. Octopuses attach a positive valence to food as ‘specializing generalists’ with long-term learning and flexible choices. They value shelter, yet modify, adapt and even transport it where necessary. They attach a negative valence to what may be described as pain, monitoring and protecting the damaged area and learning to associate locations with pain relief. Finally and surprisingly, octopuses attach a negative value to uncertainty so that they explore their environment before exploiting certain aspects of it and even exhibit motor play. This series of four papers, culminating in the present one, demonstrates in detail why the Cambridge Declaration of Consciousness has suggested octopuses might have the substrate for consciousness, although it is likely not similar to or as complex as that shown by ‘higher’ vertebrate lineages.

## 1. Introduction

Birch et al.’s [1] fourth dimension of animal consciousness is titled e-richness. For them, this represents affective experiences or ‘feelings’. Evaluating any animal’s abilities in this category produces a major difficulty. The ‘hard question’ assumes that affect needs to be reported to be known, and non-human animals (with few exceptions) cannot report their emotions or anything else about themselves. One way around this lack is to evaluate both the behavioral reactions and physiological states that would accompany affect or are reported to produce affect in humans. Another is to report on valence, or the value associated with particular situations or responses. Birch et al. [1] (p. 792) comment that “valence must be present whenever there is affect-based decision making”. In other words, affect comes when you value particular situations or sensory feedback, and by that definition, affect accompanies all choices. This cannot be true as many choices are automatic, possibly reflex responses to a narrow range of stimuli; it must be true only when choices are learned and used in future decisions or responses involving a set of actions in a complex situation. For a background on this link, it is useful to look at an investigation of human motivation as a foundation for the neural and behavioral processes that underlie our emotions. In a review, Lyon [2] suggests that valence informs an organism’s decisions about what to do next and reminds us of Damasio’s finding that reason and emotion together fuel any animal’s decision-making.

From a biological view of the decision-making process, Barrett [3] suggests that the brain (1) monitors one’s needs and current states, (2) infers causes, (3) monitors trends, and (4) decides what to do next. This assumes that both ‘needs’ and decisions have valence. Barrett further reminds us that it is all one package and that subjective feelings have accompanying neural, physiological, and behavioural changes. Such valence can be negative (as in pain) or positive (in pleasure), although a negative input such as pain, signaling actual or potential tissue damage [4], has a strong valence and is perhaps the most likely one to be seen across the animal kingdom. There is one kind of valenced situation that is not the result of simple sensory input. Again with reference to humans performing this decision-making process, uncertainty and unpredictability are stressful [5] and organisms work to reduce them by attention (focusing on what one needs to know), learning (acquiring information), and habituation (learning what not to attend to). In addition, for decision making an organism must know its current states, then its attainable states, and then compute its goal states to cope with change. It is this change that needs to be either planned for or reacted to—and remember that octopuses live in a complex and swiftly changing environment. Can they attach valence to the reduction of uncertainty?

Evaluation of positive and negative valences (what does it seek? what does it avoid?) in an octopus can thus approach evaluation of the category of emotion and begin to probe into possible affective states that help guide its behavior. Perceptual richness [6] clearly guides the input, evaluates one’s state, and predicts future actions, and organisms monitor the effects of actions to judge possible causes. Unity of the self [7] allows monitoring of one’s needs and current trends, and temporality [8] unites past experiences with future actions to predict responses. This paper adds affective valence to the three other aspects of consciousness previously covered. 

It is nevertheless difficult to understand what categories of valence can guide octopuses’ behavior. Certainly, as they are generally not social beings, they do not have positive affective experiences when affiliating with conspecifics. Yet all organisms must find nourishment, so positive valence is likely attached to (1) decision-making about food. Similarly, the soft-bodied octopus must (2) seek shelter and should attach a positive valence to finding, modifying, and attaining it. Pain (3) is a negative experience for complex organisms, including octopuses, and can be seen to have a strong negative valence. Investigating these situations will give us some foundation for understanding valence in this group. We will also examine a fourth situation, the negative value attached to (4) uncertainty and how it is resolved, for similar characteristics. 

## 2. Positive Valence

### 2.1. Valence and Food Choice

An influential book, Foraging Theory by Stevens and Krebs [9] suggested that non-human animals’ food choices were driven simply by energetics, that energy produced by the digestion of food minus energy expended on finding and preparing it predicted food choice. This simple equation, which was promptly challenged, contrasts with the varied influences known to predict human choice of food, where [10] sensory cues, learned choices, and context are all major drivers of food consumption. Despite the simplicity of the theory, energetics was often held up as the expected predictor of prey choice in any animal. The equation is much too simple for many species, including the octopuses, as research has shown abundantly since then. Prey items have valence or value attached to them, and the food-finding situation is controlled partly by availability but also by learning, sensory characteristics assumed but often not known to scientists, and other influences such as predator pressure. Energetics and its modifiers can control choices in the laboratory but the situation is not so simple in the ‘real world’ where animals make their choices. The evidence of the outcome of these complex pressures and the value that must be attached to particular prey is fairly easy to view in octopuses. They take prey to a sheltering ‘home’ and consume it there so the hard remains of food are usually though not always available for assessment.

How do we know that octopuses have valences for and make choices about what items to find and consume as food? Because hard remains of prey are easily available as middens outside octopus dens, many researchers have collected them [11,12,13,14,15,16]. All these authors noticed octopuses took a wide variety of prey species but also that the percentage of prey chosen did not equal the number of prey individuals of each species in the immediate area. What might have influenced these active choices? One variable might be ease of access. *E. dofleini* predominantly chose crabs as prey, but *Hapalogaster mertensii*, known casually as the ‘hairy crab’, was under-represented in midden remains [16]. The spiky surface is likely to make it difficult for the smooth suction cups of the octopus’ suckers to gain a grip on the crab (Astroturf is similarly repellent when placed on the upper walls of aquariums) and sponges on the surface of scallops similarly protect the host from octopod predation [17]. Chitons were very common on the subtidal rocky areas where both *E. dofleini* [16] and *O. insularis* [14] foraged yet were almost absent from prey remains. There might be a problem with the ease of removal from the rocks or penetration through the integument, or it may be that low food value is available from each prey item, but chitons seemed to have limited ‘value’ for octopuses. These examples suggest that some combination of choice and availability must drive actual consumption. A clear example of the modification of preference by availability is that of *O. bimaculatus,* who much preferred crabs over two species of *Tegula* snail in the laboratory. In the field, crabs were hardly available and so the octopuses consumed much of the more preferred of the two abundant snail species [12].

Predation on the hard-shelled bivalves offers an opportunity to evaluate the limitation of energetics in controlling prey-selection decisions, both within prey species in terms of size and across species. Penetration techniques varied. The valves of smaller and weaker bivalves such as mussels can be simply pulled apart but larger ones are drilled through the shell and poisoned to weaken the adductor muscles. As larger clams are resistant to pulling, this choice of technique is also dependent on octopus size, with bigger and stronger octopuses generally choosing bigger prey, but not always [18]. Across prey species, prey choice somewhat follows an energetic preference, with the strongest muscled clam species taken least often [19]. Yet when the clams were opened and presented ‘on the half shell’, that species was preferred, so again for choice of species, energetics, and preferences interacted. A study of selection by large octopuses among clam species [20] showed that on average the prey species selected took less ‘handling time’ and thus less energy expenditure, but post-evaluation data revealed that one of the species less often selected actually had the shortest handling time. More than effort and energetic reward were evaluated.

When penetrating bivalve and gastropod shells, octopuses first test muscle resistance and if it is large, they drill into the shell [21]. This leads to a trade-off in laboratory studies of size preference; smaller prey needs little work but yields meager rewards, and larger prey demands more work with greater return. The result was a compromise: medium- sized clams were preferred over bigger and smaller ones [22]. Another study of strategies and size looked at the choice of pulling versus drilling, and they found individuals chose a wide range of sizes at which to make the switch [23]. 

In ‘real world’ oceans, there are other influences besides energetics on prey choice that may be evaluated. Prey is first located and then prepared, and octopuses appear to use visual guidance to go to likely places and then use chemotactile search to actually find prey [14]. Smaller octopus species may calculate predator pressure, take more small prey, and be time-minimizing foragers [16], whereas much larger ones can resist predation and take larger prey, following an energy-maximizing strategy [15]. Additionally, octopuses simply prefer crabs. A carefully measured study of energy expenditure and gain [24] revealed that crabs were chosen over clams by a ratio of 4:1, yet crabs required more energy to prepare and consume and yielded less energy for the work. Octopuses prefer crabs as prey when given many different choices, consume more, and gain more weight when crab is offered in the lab [25] and this preference cannot be modified by early learning [26]. We do not know why crabs are valued, but they are.

Most of these studies looked at situations where the prey was provided, but to see the mechanism by which several influences affect prey selection, it is necessary to evaluate choices in the field and also to focus on the individual. After all, it is the individual that makes decisions. Departures from the notion of octopuses as generalist predators at the population level have already been noticed [14,15,16,21]. However, seeing the particular patterns required observation at the individual level and with frequent collections of prey remains, since shells discarded from the home are often removed by water movement or scavengers [14]. When middens were checked daily at a rich and varied site in Bonaire [27], the octopus population could be seen as generalist yet some individuals within it were specialists. This is an interesting pattern of choices because a strict specialist species could be seen to be automatically responding to a narrow set of stimuli and a generalist species to be simply taking whatever prey was immediately available. The variation amongst individuals suggests an animal without strong preferences, but rather ones based on learning, with some presumably having learned particular foraging strategies or prey availability. One individual had a ‘run’ on juvenile conch snails, for instance, which give a large energetic yield yet also require effort/learning as they need to be drilled and were found in sandy areas some distance from the rocky den location (and see [11] for a similar preference). This specialist/generalist combination showed a diverse mix of both strategies [28,29] within and across species. Interestingly, in Alaska *E. dofleini* had a very diverse diet yet further south the population was specialized—on crabs [29], suggesting specialization only when this preferred prey was abundant. Habitat richness, both across mainland and island populations [30] and at the micro level in the same area [31] still predicted the breadth of prey choice; the more species that were available, the more were taken, on average. Within the second study another variable, octopus personality [32], influenced the breadth of choice. Shy octopuses had a more narrow selection of prey species than bold ones, possibly because they explored less and discovered fewer food sources. 

So the studies of octopus prey consumption reveal valence, but they also show what a variety of influences can intervene between preference and consumption. Sensory cues are important, learned choices modify tendencies, and context including availability is vital, a set of pressures similar to that proposed for our species by Mela [10]. One preference remains unexplained: why do octopuses so prefer crabs? They may provide needed nutritional trace elements, though perhaps they taste good, as they do to us, but we have no way of asking the octopus.

Assessment of valence in food choices generally is much more sparse in the cuttlefish. Evaluation of stomach contents [33] showed diet shifts across the lifespan and led to the conclusion that common cuttlefish were generalists. Yet a couple of studies of cuttlefish cognition revealed an interesting way in which valence was expressed. First, the authors decided that animals would be willing to wait longer for access to preferred prey [34]. So they offered a choice in a variation of the so-called ‘marshmallow test’ that was featured for small children as a measure of self-control [35]. They offered preferred and non-preferred food items with a small delay for delivery of the preferred one, but as soon as either was taken, the other was withdrawn. Individual cuttlefish were able to wait for 50–130 s to gain the preferred food, showing a clear control of responses. Second, cuttlefish were offered non-preferred food in the daytime, and preferred food in the evening. Cuttlefish learned the schedule and refused the non-preferred food in the daytime when the preferred was scheduled for later delivery [36]. More interesting, if the schedule of evening food delivery was reliable, the cuttlefish were willing to wait, but if the delivery was random they took the immediate but less-preferred reward. Again as with humans [5], the cuttlefish seek predictability and respond to it, but because these cognitive tests were done in isolation from any natural history observations, we have little idea of how these abilities fit into the cuttlefish’s actual choices in nature.

### 2.2. Valence and Shelter Use in Octopuses

Because of their shell-less soft bodies, cephalopods are at major risk of predation. The benthic octopuses consequently spend little time foraging for prey [37], staying within a shelter for around 70% of their active period, thus fitting the definition of a time-minimizing forager [16]. The value of such shelter is quite simple—no shelter, no octopus survival. This is particularly true for female octopuses, which attach their eggs to a solid substrate and guard and tend them. Deep sea octopuses may cluster on the scarce solid outcropping above the soft sediment in locations such as ‘Baby Bare’ [38]. Shallow water species can congregate in similar locations and interact with actions unusual for ‘asocial’ species [39] even though, given a choice, they will not shelter near each other [40,41]. Additionally, the picture is complicated by the fact that the flexible and opportunistic octopus had preference but is also able to manipulate its environment [42,43,44] and modify it to suit its needs.

In general, octopuses will be somewhat confined to solid substrates, within which suitable shelter might be abundant [45] or somewhat limited [46]. Areas such as reef edges that offer both shelter and easy access to sandy areas nearby for hunting [47] are selected. Octopuses also seem to prefer site locations looking downward and out from shore, a ‘room with a view’ [43], maybe because they can evaluate the immediate environment before they go out hunting. Octopuses selecting ready-made shelters seem to value ones that have a volume a little more than their own and have a small aperture, as with their boneless body they can squeeze through an opening that would block competitors [42]. However, if an aperture is large, octopuses will block it off with items collected from nearby, including remains of shelled prey and small rocks [43]. Females with eggs, who no longer hunt, may completely withdraw and thoroughly block off the aperture [48]. Even though octopuses can gather tactile information indicating they are sheltered, they also can see outside and prefer shelter that is dark. 

Although they need solid shelter, many octopus species can use natural shelter available far from solid rock, such as molluscan shells. The availability of empty mollusc shells enlarged the range and shaped the distribution of *O. joubini* on sea-grass beds and enrichment of the area with gastropod shells increased octopus density [43]. Such enrichment by artificial shelters can rejuvenate a population that has been over-fished by providing shelter for brooding females [49]. Oyster beds far from solid rock offered empty shells to shelter *O. tehuelchus* [50], a scallop bed gave shelter for an unusually crowded group of *O. tetricus* [39] and a variety of human-made structures allowed *O. vulgaris* to survive on soft sediment [44]. All of these observations indicate the imperative need for some kind of shelter, but the fact that they can modify physical structures makes it difficult to specify what type of shelter an octopus ‘values’. 

One of the few positive results of pollution of the marine environment is the widespread use of glass, plastic, and metal waste for octopus shelter [51,52,53], sometimes allowing them to live in ‘urbanized’ seascapes [54]. Dark beer bottles are ideal for *O. rubescens* [55], tires for *O. vulgaris* [44]. Split coconut shells are light enough to be portable so that *O. tetricus* can imitate hermit crabs and actually take shelter with them when they forage on the sandy/mud substrate [56]. Perhaps the value of shelter is best demonstrated by this, how much the octopuses work and adapt their environment to attain it.

## 3. Negative Valence

### 3.1. Valence and Pain

Pain, defined as “an unpleasant sensory and emotional experience associated with actual or potential tissue damage” [4], is based on a universal sensory input across the animal kingdom yet lifted to the experiential level by central monitoring, evaluation, and decision-making. The sensation is vital and imperative, the signal amount increasing swiftly with more potential damage and the input habituating poorly. Yet the central monitoring matters, as humans without pain sensation cannot self-monitor damage and do not live long lives. Pain is thought to have sensory, cognitive, and affective components in humans and thus it is impossible to absolutely prove its existence in non-human animals. There is a widespread debate about whether and which animals can experience pain, as those with simpler nervous systems are often designated as having nociception, solely receiving the sensory component. Sneddon et al. [55] acknowledge this problem and suggest that many pieces of evidence can be accumulated to make a good case for true pain and thus valence in non-human animals. They suggest that there should be a neurobiological, physiological, and behavioral response to a noxious event, that this should result in avoidant and protective responses afterward, and that we should see subsequent changes in motivational state such as place preference and either analgesia self-administration or that the animal pay an energetic cost to access it. 

Besides the obvious tissue damage, many marine animals presumably experience nociception from the sting of nematocysts (also sensed by humans) of the phylum Cnidaria [56]. Octopuses show signs of aversion after contact with cnidarian sea anemones, and hermit crabs place anemones on their sheltering gastropod shell to protect themselves again predators including octopuses [57]. After being stung, octopuses try different approaches to an anemone-carrying crab, such as coming from a different angle or trying to blow the anemone off the crab with jets of water. This avoidance is only partly successful, but observation of aversion to stings gave the early researchers looking for a learning situation in octopuses the idea to use a small electric shock in aversive conditioning [58]. 

Whether it was from chemical or mechanical stimuli, researchers have slowly accumulated evidence that cephalopods have more than simple nociception. A complex set of responses takes place if squid are given a small injury and then exposed to a potential predator [59]. First, and notably, a sea bass recognized some behavioural or chemical differences about a squid that had been injured and was more likely to attack it. The injured squid changed their behavior and became wary. They were alerted to the predator from further away and sooner in its approach and fled sooner (note that loliginid squid in midwater are ‘jumpy’, moving away from a potential threat on average eight times per hour [60]). The injured squid had a heightened sensitivity to mechanical stimuli, but if they were given anesthetic, they lost this sensitivity and wariness and were more likely to be captured by the fish. While squid thus have a heightened response to damage, the authors did not see any behavioral response to the wound itself. However, this study was only short-term.

Octopuses given a similar arm crush injury showed their normal aversive behavior of inking, jetting away, and, in the case of *Abdopus*, arm autotomy [61]. Their sensitivity to mechanical touch increased, both locally around the damaged area and more generally across the skin surface. Local mechanoreceptors were sensitized and twenty-four hours later they still had a lower threshold but on the ipsilateral though not the contralateral arms. There was a local seeking response of nearby suckers towards the damaged area, as if exploring for the source of the problem (a protective response). This sensitivity increase was abolished with anesthetic. Interestingly, octopuses showed immediate arm grooming near the injury and persistent arm guarding, criteria expected as part of affective responses [55]. A different octopus species, *O. bocki,* was given a presumably painful injection of acetic acid in the arm and then confined in a previously positively conditioned chamber [62]. The experience led octopuses given the injection to now avoid this chamber. If, however, they were given a lidocaine injection that relieved the sensation, they stayed in what had now become a rewarding location. Both of these place responses fulfil the motivational changes expected to demonstrate likely sentience [55] so we can recognize that damage has the expected negative valence. 

### 3.2. Valence and Lack of Information

There is one surprising aspect of cephalopod behavior which has been studied mostly in octopuses, that a positive value is attached to gaining information. It is not one that we normally think of as part of animals’ lives, mainly because studying details of behavior in a controlled and constrained laboratory limits its expression. Additionally, Peters [5], focusing on humans from a biological standpoint, pointed out that uncertainty (unexpected novelty, unpredictability, and uncontrollability) is stressful and that organisms act to reduce this uncertainty. In that sense, knowledge of the environment has a positive valence. He suggested that three processes reduce uncertainty—attention, learning, and habituation—and that in mammals, glucocorticoids in the brain are central to these processes. In rats, there is a clear effect of environmental enrichment on the reduction of anxiety as well as an increase in exploratory behavior, again linked to glucocorticoid receptors in the brain [63]. The value of this uncertainty reduction can be seen from two different viewpoints. One is motivational, in that researchers have found that mobile animals always explore a new environment, expressed in terms of orienting to situations of interest, manipulation of items nearby, and locomotion through the area [64]. It might be a basic drive to explore or one to increase the amount of sensory information available. Separately, information seeking might be linked to ecological necessity, a need to balance exploration (gaining information) with exploitation (using the information to satisfy basic needs) [65]. The authors suggest that animal monitoring of this balance might be a simplistic form of the metacognition that we humans use.

There are two spatial referents in which the balance is expressed, reflecting the division between close-by egocentric and larger allocentric space [8]. In egocentric space, animals explore their immediate environment, probably to reduce the uncertainty about what is around them [5]. Again, object manipulation is linked across primate species to being a generalist feeder with a diversity of food handling procedures [66], and remember that octopuses are also generalists. It also appears to be linked to brain size [67]. This kind of object manipulation may be a foundation for the cognitive skill of mental manipulation [68], one of the bases for human sentience. While the exploration of one’s immediate environment is widespread, it extends in some species into two different special activities, tool use and play. Although tool use was once described as discriminating humans from non-human animals [69], simple tool use, defined as “the exertion of control over a freely manipulable external object with the goal of altering the physical properties of another object, substance, surface or medium via a dynamic mechanical interaction” [70] is now known to be much more widely distributed in the animal kingdom. It is thought to be more sophisticated in mammals, especially primates, and corvid and parrot bird groups. The use of tools may actually extend the egocentric body space [71] and the tool can be included in the immediate body schema and change neural networks in the process. Such actions must be planned and the goal has a positive valence in some way.

The aquatic environment is not always supportive of object manipulation and tool use, with the lack of objects except on the benthos and the higher density of water making object movement more difficult [72]. As well, we lack information about marine animals because the oceans are poorly explored. On the other hand, water itself may be a tool, and cephalopods have used jets of water aimed through the flexible funnel for many different functions. Octopuses [73], cuttlefish [74] and sepiolid squid [75] manipulate the substrate, particularly sand, to form hiding places in crevices or underneath a sandy surface. Octopuses also use jets of water to repel scavenging fishes [76] and occasional pesky experimenters, and finally in object play [77]. This manipulation is a prime example of domain generality, extending the use of behavior that originally evolved as circulation of water through the mantle cavity for respiration, to jet propulsion, and into object propulsion then apparently to play, an excellent example of octopuses’ behavioral flexibility.

A second category of manipulative behavior that is thought to have evolved from exploration is play. Except for having large complex brains and a demanding environment, cephalopods do not fit the category of animals that are presumed to play. Play consists of incomplete or out-of-context action, is not stereotypical, is produced spontaneously in a stress-free situation, and is not obviously ‘useful’ [78]. Normally play is seen in mammals. It is thought to arise because young mammals have excess energy resources, a sheltered juvenile period that gives them time to express play, and because it is useful, i.e., has positive valence for preparing them for adult lives, particularly in social roles. Some groups such as the parrot play through adulthood, perhaps to cope with a varied environment and to practice generalist foraging strategies [79]. We can think of exploration as finding the affordances (potential ecological roles) of objects in the environment, and object play as manipulating those affordances. Octopuses, being asocial, do not perform social play, but do play with objects using different actions. The first play behavior recorded used the water jet, moving a floating object around an aquarium [77], and the second was moving an object by one or more of the mobile arms [80]. A third context has been casually reported, where a floating object is pulled underwater and allowed to bob back up again. As in tool use, the octopus has a great deal of behavioral flexibility in play. Yet play is defined as having a hedonistic character in that it is ‘pleasurable’, while exploration is assumed to reduce anxiety [67] so by definition these are valenced actions.

Uncertainty is also resolved by exploration in the larger allocentric environment, as mobile animals need to know in particular what resources are in the areas into which they will move. Forging bees balance exploration of areas with potential resources with exploitation of these resources, beginning with short trips near the hive, moving to longer exploratory ones, and then to directed exploitation by traplining [81]. Rodents establish a ‘home base’ and first make short excursions from it, later making longer ones and using a saltatory stop-and-go pattern of movement [82] and this pattern is also found in octopuses [14]. Yet rats also shift their routes depending on the certainty of the potential rewards along them [83]. Exploration is linked to diet, with frugivorous primates exploring less and having simpler paths than generalists [84]. It seems also tied also to the demands of a complex environment across their life history since corvid birds explored and played as juveniles and parrots throughout their lives [79] and remember that octopuses live in a diverse and changing environment. Given a novel laboratory environment, octopuses spent much of their first 24 h exploring, and this activity decreased with time [85]. They learned the location of sheltering burrows during exploration and used them when they were needed later, increasing their movement again when the testing arena was rotated and locations had to be re-calibrated. Exploration is part of the normal lives of cephalopods although it is very poorly documented, and its reduction is likely valenced. 

## 4. Conclusions

The four papers of this series have attempted to look at the foundation for sentience in the cephalopod mollusc octopuses and have evaluated as much as presently possible their ‘consciousness profile’ [1]. Cephalopods have rich perceptual experiences [6], but the dimension of these experiences are not necessarily the same as the mammals we are related to and think in terms of. Despite the decentralized distribution of the nervous system, with many neurons in the octopod arms, cephalopods have a basic unity of evaluation and a central brain that learns and makes decisions [7]. They make and use these decisions across time, from monitoring their needs and inferring causes to monitoring trends [3], before deciding on and carrying out actions (see Neisser [86] for this process in humans). Rather than simply seeing this ability as an accumulation of ‘unlimited associative learning’ capacities [87], we may need to evaluate the patterns of attention, learning, and habituation [5] that are necessary to live in a complex world. It is more than acquiring bits of information to use immediately. In fact, picking up a sheltering coconut shell to carry for future shelter [54] as well as choosing to reject unpreferred prey due to the certainty of later preferred prey delivery [36] come close to the ‘mental time travel’ [1] suggested as evidence for consciousness.

Much of the evidence for a basis for sentience in these complex animals comes from evaluating valence, what ‘matters’ to animals. The multiple influences on food choice contradict the simplistic energy tradeoff model [9] and leave us wondering whether cephalopods may actually like some foods more than others. Shelter is vital to the soft-bodied octopuses, but they do not choose ready-made spaces, instead manipulating objects, again suggesting that they have some kind of mental template [3] of what they need. Parallels with human valences can be useful in testing for animal sentience. As Sneddon et al. [55] suggest, behavioral parallels in cognition lead us to conclude that animals such as cephalopods may have subjective pain. Similarly, parallels between humans and cephalopods in the exploration/exploitation processes may suggest a need to reduce the uncertainty about the world around them which is stressful for humans [5]. This pattern of information acquisition seems to lead to play and again may be evoked by living in a complex and varying environment [79]. While we can never ‘prove’ that any species has sentience, the information presented in this series of papers clearly points out that the foundation for this capacity is present in cephalopod molluscs such as octopuses.

## Data Availability

Not applicable.
